# Toxoplasmosis: Epidemiological Significance, Economic Burden, and Emerging Directions in Drug Discovery and Artificial Intelligence

**DOI:** 10.1155/ijm/3271467

**Published:** 2026-10-14

**Authors:** Albert Fynn Aiduenu, Blessing Kwabena Gayi, Aboagye Kwarteng Dofuor, Susanna Awinsongya Anovunga, William Ekloh, Joseph Harold Nyarko Osei, Christopher Nii Laryea Tawiah-Mensah, Juliet Ama Mawusi Koudonu Seglah, Jennifer Afua Afrifa Yamoah, Angelina Akomea, Andy Asafu-Adjaye

**Affiliations:** ^1^ Department of Biochemistry Cell and Molecular Biology University of Ghana Accra Ghana ug.edu.gh; ^2^ West African Centre for Cell Biology of Infectious Pathogens University of Ghana Accra Ghana ug.edu.gh; ^3^ Department of Biological Sciences School of Natural and Environmental Sciences University of Environment and Sustainable Development Somanya Ghana uesd.edu.gh; ^4^ Department of Environmental Toxicology Southern University and A&M College Baton Rouge Louisiana USA subr.edu; ^5^ Department of Biochemistry School of Biological Sciences College of Agriculture and Natural Sciences University of Cape Coast Cape Coast Ghana ucc.edu.gh; ^6^ Department of Parasitology Noguchi Memorial Institute for Medical Research College of Health Sciences University of Ghana Accra Ghana ug.edu.gh; ^7^ Biomedical and Public Health Research Unit Water Research Institute Council for Scientific and Industrial Research Accra Ghana csir.org.gh; ^8^ Department of Molecular Biology and Biotechnology University of Cape Coast Cape Coast Ghana ucc.edu.gh; ^9^ Animal Health Division Council for Scientific and Industrial Research-Animal Research Institute Accra Ghana; ^10^ Department of Pharmaceutical Sciences University of Oklahoma Health Oklahoma USA

**Keywords:** artificial intelligence, epidemiology, *Toxoplasma gondii*, toxoplasmosis

## Abstract

Toxoplasmosis, a parasitic disease caused by *Toxoplasma gondii*, affects a wide range of warm‐blooded hosts and remains most prevalent in tropical regions, with distribution shaped by climate, cultural practices, and environmental conditions. Clinical severity varies across susceptible populations, generating substantial economic burdens through medical costs and agricultural losses. Given this global health impact, a comprehensive, up‐to‐date understanding of the disease is essential. However, significant gaps persist. While diagnostic techniques and epidemiological surveillance have advanced considerably, treatment protocols have remained largely unchanged for decades, revealing a disconnect between diagnostic innovation and therapeutic progress. Moreover, existing literature lacks an integrative synthesis connecting these advances to their broader significance, leaving researchers without a unified framework for understanding where the field stands and where it should go next. To address these gaps, this narrative review draws on peer‐reviewed literature and institutional reports across major biomedical databases, synthesizing findings descriptively and thematically rather than through statistical meta‐analysis. It critically examines toxoplasmosis across several dimensions. It first considers geographical distribution and the cultural, environmental, and economic factors shaping its epidemiology. It then turns to the parasite′s biochemical and molecular characteristics, using this foundation to evaluate persistent limitations in treatment and vaccine development, an area where progress has stalled. In response, the review explores emerging alternatives, including plant‐based compounds, as avenues beyond conventional pharmacological strategies. Finally, it examines artificial intelligence as an underexplored but promising tool for improving diagnosis and treatment, an area with limited application to toxoplasmosis specifically. By consolidating these threads, this review clarifies not only what is known about toxoplasmosis but also where critical gaps in treatment innovation and integrative understanding continue to hinder effective prevention and control.

## 1. Introduction

*Toxoplasma gondii* is the protozoan parasite that causes zoonotic toxoplasmosis [1]. The phylum of *Apicomplexa* intracellular coccidian parasites includes this species [2]. Its nuclear genome, approximately 65 million base pairs in size, has been reported from a karyotype of about 13 chromosomes [3, 4]. According to McFadden and Reith [5], discovering the apicoplast (an organelle resembling a plastid with a 35 kb episomal genome) unveiled similarities to photosynthetic organisms. The fatty acid synthesis and heme biosynthesis metabolic pathways of *T. gondii*, which are essential for the parasite′s survival and pathogenicity in a variety of hosts, are based on this genome structure [6, 7].

Domestic pets and livestock are usually responsible for spreading *T. gondii* [8]. The parasite rarely causes clinically significant disease despite infecting a significant portion of the world′s population [9]. It has received considerable attention due to its extensive distribution and significant impact on human and animal health [1]. Since its discovery in the early 20th century, researchers have been intrigued by the parasite′s remarkable adaptability and longevity in a variety of climates and ecosystems, which has led to its classification as one of the most prevalent parasites worldwide, with up to one‐third of the human population being estimated to be infected [10].

Toxoplasmosis typically manifests as a mild or asymptomatic *Toxoplasma* infection in immunocompetent people; however, in immunocompromised patients, it can lead to severe complications such as encephalitis, ocular disease, and systemic infections [11, 12]. Toxoplasmic chorioretinitis is the most prevalent clinical sign of *T. gondii* infection in immunocompetent people. It usually manifests as unilateral or bilateral retinal inflammation, resulting in symptoms like blurred vision, floaters, and eye pain [13, 14]. Characteristic necrotizing retinochoroiditis lesions are the result of either a newly acquired infection or the reactivation of a congenital infection [15]. Recurrent episodes may result in permanent vision impairment, although the majority of cases are self‐limiting [16].

Globally, there are significant variations in the disease′s prevalence [17, 18]. With an estimated 1.2 million disability‐adjusted life‐years (DALYs) for over 190,000 annual cases, congenital toxoplasmosis (CT) continues to pose a significant threat to global health [19, 20]. Untreated *Toxoplasma* infections in children may result in long‐term neurological sequelae and visual impairment. HIV‐associated *Toxoplasma* reactivation is still very common in low‐income countries despite quickly declining in high‐income countries following the introduction of highly active antiretroviral therapy. It is reported that over 13 million HIV‐positive individuals worldwide are seropositive for *Toxoplasma*, putting them at risk for cerebral toxoplasmosis; 87% of these individuals reside in sub‐Saharan Africa [21]. Thus, toxoplasmosis has a significant financial impact on the agricultural and healthcare industries.

The parasite′s capacity to create tissue cysts enables it to live in the host for protracted periods, aiding its spread as a pathogen [22]. Felids are the only known definitive hosts for this parasite; when infected felids spread, they excrete millions of oocysts in their feces, which sporulate and spread throughout the environment to become infectious [23]. Feral cats may occasionally experience oocyst shedding throughout their lifetime, even though it only occurs 1–3 weeks after the initial *Toxoplasma* infection in domestic cats [24]. The two main horizontal transmission routes are the consumption of tissue cysts containing bradyzoites from raw or undercooked meat or ingesting these sporulated oocysts, which infect plants, soil, and water supplies [25]. Another route of transmission of the parasite may be organ transplantation or blood transfusion from infected people [26]. Thanks to advancements in detection methods, including imaging techniques, polymerase chain reaction (PCR)–based methods, and serological tests, the disease is diagnosed easily [27]. Currently, the diagnosis of human infections caused by *T. gondii* is largely dependent on serological testing [28].

There have not been many advancements in toxoplasmosis treatment in epidemiology or understanding of parasite–cell interactions. Antiparasitic medications such as pyrimethamine and sulfadiazine are the mainstay of current treatments for toxoplasmosis; however, their effectiveness is frequently restricted by side effects and the possibility of resistance [29]. Although there has been progress in developing vaccines, especially in animal models, a completely effective human vaccine is still unattainable [30]. Remarkably, recent research on *T. gondii* has examined the effects of plant‐based compounds, emphasizing the potential of natural products as complementary or alternative therapies [30, 31]. Furthermore, the use of artificial intelligence (AI) in toxoplasmosis detection and treatment is still in its infancy, but AI‐driven models have the potential to improve diagnostic precision and forecast treatment outcomes [32].

Toxoplasmosis continues to be a serious global health issue with far‐reaching effects on economies, agriculture, and public health. Despite significant advancements in our knowledge of the biology of the parasite and the creation of diagnostic instruments, significant improvement in treatment and prevention strategies is still required. The present review is aimed at providing a practical understanding of the disease through an integrative and critical approach. We highlight the economic impact, epidemiological implications, molecular characterization, drug discovery, current vaccine status, and AI applications for toxoplasmosis.

## 2. Methodology

### 2.1. Aims and Objectives

This review was designed to provide an integrative, up‐to‐date synthesis of *T. gondii* and toxoplasmosis across five interconnected dimensions: geographical and epidemiological distribution, biochemical and molecular biology, economic burden, drug and vaccine development, and emerging plant‐derived and AI‐based approaches to diagnosis and treatment. The specific objectives were to (i) map the global and regional epidemiology of toxoplasmosis in relation to cultural, environmental, and health‐system factors; (ii) characterize the parasite′s biochemical and molecular features relevant to pathogenicity and drug targeting; (iii) evaluate the economic impact of the disease on healthcare and agricultural sectors; (iv) assess the current state of, and persistent stagnation in, drug and vaccine development; (v) appraise the evidence for plant‐derived compounds as alternative antiparasitic agents; and (vi) examine the underexplored potential of AI in improving toxoplasmosis diagnosis and treatment. Collectively, these objectives are aimed at consolidating fragmented literature into a single framework identifying where knowledge is well established and where critical gaps remain.

### 2.2. Research Approach

This is a narrative, integrative literature review. This approach was chosen because the review′s scope spans multiple disciplines—epidemiology, biochemistry, pharmacology, ethnobotany, and computational science—that are not well served by a single systematic protocol or homogeneous outcome measure suitable for statistical pooling. The review synthesizes findings thematically across sections, drawing connections between historical, clinical, economic, and technological literature that are rarely discussed together in a single source.

### 2.3. Literature Databases and Search Strategy

Literature was retrieved from major biomedical and life‐science databases, including PubMed/MEDLINE, Scopus, Web of Science, Google Scholar, and ScienceDirect, supplemented by targeted searches of parasitology‐specific and veterinary databases (e.g., CAB Abstracts) and institutional or organizational reports (e.g., WHO) for epidemiological and economic burden data. Reference lists of retrieved articles and prior reviews were also hand‐searched to identify additional relevant sources not captured by the primary search strategy. Approximately 10 key search terms and their combinations, using Boolean operators (AND/OR), guided the search: *T. gondii*, toxoplasmosis, epidemiology, CT, economic burden, drug development, vaccine development, medicinal plants/plant‐derived compounds, AI, and machine learning/deep learning diagnostics.

### 2.4. Inclusion and Exclusion Criteria

Articles were included if they were peer‐reviewed original research articles, reviews, or institutional reports published in English; reported primary or synthesized data on *T. gondii* biology, transmission, epidemiology, economic impact, treatment, vaccine development, plant‐derived antiparasitic compounds, or AI applications in parasitic disease diagnosis and management; and were accessible in full text. A small number of foundational historical papers dating to the early‐to‐mid 20th century was included where necessary to contextualize the discovery and early clinical characterization of the parasite. Articles were excluded if they were non‐peer‐reviewed (e.g., blog posts, nonindexed preprints, and opinion pieces without data), duplicates across databases, available only as conference abstracts without full text, unrelated to *T. gondii* or toxoplasmosis despite keyword overlap, or published in a language other than English without an accessible translation. Studies of markedly low methodological quality or from predatory journals were also excluded.

### 2.5. Study Selection and Data Extraction

Search results were screened first by title and abstract, then by full‐text review, to determine relevance to the review′s thematic sections. Data extracted from each included source were organized according to the section of the review to which they were most relevant (e.g., transmission biology, molecular characteristics, economic modeling, drug/vaccine trials, phytochemical studies, or AI applications) rather than into a single standardized extraction template, consistent with the narrative review design.

### 2.6. Publication Scope and Volume

While a number of classic references are retained for historical context on the discovery and early clinical description of toxoplasmosis, the majority of cited literature was published after 2015, ensuring the review reflects contemporary understanding of diagnostic, therapeutic, and technological developments.

### 2.7. Data Synthesis and Analysis

Data were analyzed descriptively and qualitatively. Findings were grouped thematically, compared across geographic regions and host populations where relevant, and synthesized to highlight consistencies, contradictions, and gaps in the literature. Particular attention was paid to identifying the disconnect between advances in diagnostic and epidemiological methods and the comparative stagnation in treatment and vaccine development, which forms a central thread connecting the review′s later sections on plant‐derived alternatives and AI‐based innovations.

## 3. Brief Historical Overview of Toxoplasmosis

In the absence of direct evidence from fossil records, genetic studies suggest that the genus *Toxoplasma* may have existed about 10 million years ago. Toxoplasmosis has also been suggested historically to have existed in antiquity, with an estimated existence associated with that of cats in ancient times [33]. Evidence from genetic analyses suggests that clonal lineages emerged about 10,000 years ago after a single genetic cross. This timeline is in agreement with Azab′s estimated time for the existence of toxoplasmosis. The clonal lineages of *Toxoplasma* species have been successful. Their success had been attributed to their ability to directly infect their host via oral routes. This had been substantiated by comparing these clonal lineages with ancient strains approximately 1 million years old. It has been speculated that the existence of *T. gondii* coincided with the domestication of agricultural and companion animals [34]. This started in the Near East during the Neolithic era around 12,000 years ago [34]. The tachyzoites of the parasite were found in an intermediate host called *Ctenodactylus gundi* at the Pasteur Institute in Tunis [35], which was used for leishmaniasis research [36]. In the 1900s, Charles Nicolle accidentally discovered *T. gondii*, initially misidentified as a piroplasm but later correctly identified as *T. gondii* [37] while searching for reservoirs of *Leishmania* parasites. A similar parasite was also independently found in a rabbit by another investigator in Sao Paolo, Brazil, Alfonso Splendore [38]. This prompted the scientific characterization and vigilance for *Toxoplasma* and *Toxoplasma-*like organisms between 1908 and 1937 [39].

In 1909, Nicolle and Manceaux proposed the name *T. gondii* based on its bow‐shaped (toxon) infectious stage and a possible connection to the gundi rodent. Initially considered related to *Leishmania*, its significance as a human pathogen was not recognized. A detailed study in 1937 by Sabin and Olitsky identified *T. gondii* as an obligate intracellular parasite transmissible via tissue homogenates. They noted variations in virulence and the presence of neutralizing antibodies in monkeys [40]. In 1937, human *Toxoplasma* infection was documented in an infant [41, 42]. The first clinical manifestations of CT were reported in an infant in 1939 [43]. Later studies confirmed congenital transmission from asymptomatic mothers [44]. The typical sign of toxoplasmosis was later described in 1942 [45]. By the 1940s and early 1950s, fatal cases in adults and ocular toxoplasmosis were reported, establishing *T. gondii* as a cause of severe disease [46, 47]. Around this same period, serological advancements revolutionized the diagnosis of *T. gondii*. The Sabin–Feldman dye test was used to detect *T. gondii* antibodies on a large scale [48]. This revealed widespread prevalence in both animals and humans. Also, several drugs were identified by working groups to be effective for the treatment of *T. gondii*. These drugs included sulfonamides for the treatment of murine toxoplasmosis, sulfonamides and pyrimethamine combination therapy for the treatment of human toxoplasmosis, spiramycin for the treatment of pregnant women and mice, and clindamycin as an alternative for patients allergic to sulfonamides [45, 49, 50]. The disease had a grave impact on agriculture, particularly abortion in sheep, leading to global economic losses [51, 52]. Hutchison in 1965 linked *T. gondii* transmission to cat feces [53]. In 1965, Desmonts et al. also demonstrated transmission through undercooked meat, which was a significant mode of human *Toxoplasma* infection [54]. Later, in 1970, Hutchison discovered the sexual phase in cats. Therefore, from the 1960s up to the 1970s, the life cycle and transmission of *T. gondii* were clearly and completely elucidated. Environmental transmission routes were also illustrated following outbreaks in marine mammals [55].

The acquired immune deficiency syndrome (AIDS) epidemic in the 1980s highlighted toxoplasmosis as an opportunistic *T. gondii* infection, emphasizing its clinical impact in immunocompromised individuals [37]. From the 1980s up to the 2000s, genetic variability among *T. gondii* isolates was recognized. The role of various hosts and transmission routes, such as oocysts and meat, among others, was extensively studied through epidemiological research [37]. Advances in molecular methods such as PCR also made the detection or diagnosis of the disease very reliable. Burg et al. were the first to detect *T. gondii* via PCR while targeting the B1 gene of the parasite.

## 4. Transmission Profile of Parasite

The disease has a complex life cycle and can be transmitted through a variety of hosts and pathways. *T. gondii* may infect both terrestrial and aquatic mammals as well as birds as hosts (Figure 1). These animals are referred to as intermediate hosts because they only experience asexual stages of the parasite′s life cycle [56]. The only exception to this is the family Felidae, including domestic cats, which can experience the sexual phases of the parasite′s life cycle [57]. They are therefore regarded as permanent or definitive hosts. However, recent research has found that giving mice supplements of linoleic acid and inhibiting the activity of an enzyme called delta‐6‐desaturase in their intestines can allow *T. gondii* to experience sexual growth in the intestinal cells of mice [58].

**Figure 1:**
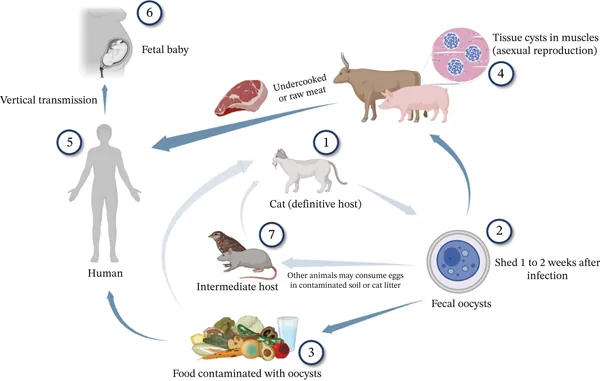
Transmission and life cycle of *Toxoplasma gondii*. (1) Cats act as definitive hosts of *Toxoplasma gondii*, where sexual reproduction, the enteroepithelial cycle, and production of oocysts occur. (2) An infected cat releases oocysts in their feces after 1–2 weeks of infection. Oocysts sporulate and become infective within 5 days after release into the environment. (3) Food and water become contaminated upon contact with oocysts. (4) Other animals such as pigs and cattle may consume parasitized eggs in the soil, water, or on plant material. Upon consumption, the eggs release motile forms of the parasite called tachyzoites, which migrate through the body of the animal and form cysts in muscle and nerve tissues via asexual reproduction. (5) Humans become infected by consuming raw or undercooked meat contaminated with tissue cysts or by eating foods or water contaminated with fecal oocysts. (6) Congenital or vertical transmission has also been observed, and this occurs during the acute phase of toxoplasmosis. Tachyzoites cross the placental barrier and cause infection in the fetus. (7) Cats can become infected by consuming contaminated prey such as rodents and birds. The authors created the diagram, including all arrows and labels.

The *T. gondii* life cycle comprises three distinct developmental stages that correspond to various phases of infection and host interaction: (i) an invasive tachyzoite, which is characteristic of the acute phase of infection; as the infection progresses and due to immune pressure or stress, they transform into a (ii) bradyzoite, which is characteristic of chronic *T. gondii* infections. Bradyzoites are typically found within tissue cysts in the host′s muscles and brain. They are metabolically quiescent and can persist in the host for extended periods, evading immune detection [59, 60] (iii) sporozoites, which are found in the environment [23].

The sporozoite form of life, which is an environmental stage, is shielded by a multilayered oocyst wall, thereby protecting the complex structure from chemical and mechanical stress, allowing the parasite to survive for more than a year [61]. The highly active tachyzoites can infect and replicate in many different types of cells in vertebrates, where they hijack the host cellular machinery, leading to extensive damage [62]. The immune system attempts to contain the proliferation, which results in inflammation and cell death. The tachyzoites disseminate through the lymphatic system and bloodstream, infecting various tissues such as the brain, eyes, and muscles [63, 64]. Following a period of multiplication in a week or two, the immune response becomes more effective in controlling the tachyzoite population. They can transform into bradyzoites, which form tissue cysts and are typically larger in muscle cells and smaller in brain cells [1]. These cysts contain a high number of tightly packed bradyzoites, which have a protective outer layer that allows them to survive for long periods of time and can resist being broken down by the host′s digestive enzymes [65]. The nature of bradyzoites allows perfect adaptation for long‐term survival, and the cysts can persist in cells of the host. They are resistant to the pepsin enzyme produced by the host, allowing them to persist for 1–2 h [1]. Bradyzoites are also not responsive to antibiotics [66]. In a healthy host, there can be conversion of bradyzoites back into active tachyzoites, making it possible for the immune system to destroy them.

Infection routes of toxoplasmosis include the ingestion of contaminated milk and dairy products containing tachyzoites, consumption of raw lamellibranch mollusks, and fresh products contaminated with sporulated oocysts from cat feces or contaminated water [67].

As already noted, only members of the Felidae family undergo the sexual cycle of *T. gondii* [23]. The most common way cats acquire a *Toxoplasma* infection is by the ingestion of prey that has tissue cysts or oocysts that contain sporozoites from another feline [57]. Upon ingestion, the cysts rupture due to the action of proteolytic enzymes and the stomach′s acidic environment, releasing bradyzoites or sporozoites depending on the cyst type [57]. Regardless, the first invaded cells will be intestinal epithelial cells, which will thereafter transform into schizonts, an asexual reproduction stage characterized by the presence of numerous nuclei [23]. Several nuclear divisions during schizogony lead to the development of individual cells. The apical complex, which includes micronemes and rhoptries, all characteristic components of a typical Apicomplexa, may be seen concurrently with the formation of an inner membrane complex needed for each merozoite [23]. Inside the host cell, these cytoplasmic structures arrange around each nucleus to produce the daughter cells referred to as merozoites. Many merozoites are released when these enterocytes burst, and these merozoites are known to infect new enterocytes with subsequent division by schizogony [57]. The quantity of merozoites produced by each schizogonic cycle increases rapidly, allowing them to readily infiltrate new enterocytes. The schizonts and merozoites are mostly detected in the ileal part of the gut 3–15 days after the feline initial *Toxoplasma* infection, and several of them start to develop into gametes [23]. One merozoite produces one macrogamete. The immature oocyst is created by the fusion of a microgamete and a macrogamete and is discharged into the intestinal lumen of the final host. The interior of the immature oocyst, which has an oval shape, houses a single sporoblast. Once the oocysts have been released in the cat′s excrement, the aerated atmosphere causes maturation, which results in sporulation that occurs within a short time frame (36 h) [23]. The oocyst separates into two sporocysts during sporulation. Each sporocyst contains four sporozoites after the oocyst matures later. The sporulated oocysts are extremely resistant to environmental factors, can survive for several months in both wet and dry conditions, and can then be ingested by other mammals [68].

Intermediate hosts can be infected with *T. gondii* through the ingestion of sporulated oocysts, tissue cysts, and tachyzoites. It should be noted that tachyzoites are the most important stage in terms of epidemiology [69, 70]. Upon *Toxoplasma* infection in intermediate hosts, the sporozoites and bradyzoites of *T. gondii* differentiate into tachyzoites, undergo replication inside a parasitophorous vacuole, and spread rapidly through the host′s tissues. The tachyzoites can differentiate into bradyzoites, which form tissue cysts that can remain viable in the host for a long period of time. Tachyzoites are responsible for acute infection and spread of the disease, whereas bradyzoites cause chronic infection without noticeable symptoms. The ability of *T. gondii* to interchange between tachyzoite and bradyzoite forms allows it to survive in intermediate hosts and to reactivate infection when the host′s immune system is no longer able to control tachyzoite proliferation [23]. In humans, these cysts develop in neural and muscular tissues, specifically, in skeletal and cardiac muscles and the brain ([57]; [71]). They can remain dormant in the host′s body for years until the host is consumed by a predator or a new cycle begins. However, in immunocompromised individuals, the bradyzoites can be released from the cysts, potentially causing acute encephalitis [72, 73].

The complex protozoan parasite exhibits antigenic diversity that has led to ongoing challenges in the treatment and diagnosis of toxoplasmosis. Recent studies have shifted focus from the known moving‐junction complex to the glideosome, which is made up of unconventional myosins and associated proteins believed to play a vital role in parasite motility and the invasion process into host cells [74, 75]. The glideosome in *T. gondii* facilitates host cell invasion by connecting actin filaments to surface adhesins, enabling myosin‐driven force transmission. This mechanism allows the parasite to glide over substrates, effectively penetrating host cells and overcoming biological barriers [76].

## 5. Biochemical and Molecular Significance of *Toxoplasma*

In addition to its worldwide distribution and highly diverse host range, *T. gondii* displays great genetic diversity, which is partly responsible for the variability in infection severity. *T. gondii* strains are traditionally classified as “clonal” or “atypical” according to their genetic characteristics. Recent studies targeting microsatellite sequences showed that although so‐called clonal strains share genotypic markers, they differ at the fingerprint level. Because they are not actual clones, the term “archetypal strains” has also been proposed instead of clonal strains [77, 78]. Additionally, novel *T. gondii* genotypes have been designated as exotic or recombinant based on their allelic composition [79, 80]. With a genome size of approximately 65,464,242 bp (~65 Mbp) (ToxoDB‐48TgME49 reference genome), the final polished and scaffolded assemblies were shown to consist of 13 chromosomes [4].

Conventional designation defined the clonal Type I, II, and III lineages, lumping all others together as atypical or exotic genotypes [81, 82]. Several molecular techniques, including polymerase chain reaction–restriction fragment length polymorphism analysis (PCR‐RFLP) [83, 84], microsatellite DNA analysis [77, 85], and multilocus DNA sequence typing of introns [86, 87], have been used to study the genetic makeup of *T. gondii* strains. Subsequently identified major genotypes were added to this list, including Type BrI, BrII, BrIII, and BrIV; Type 12; Africa 1; and Chinese 1 [86, 88, 89]. Other groups defined by microsatellite typing include Caribbean 1, 2, 3; III Variant; Amazonian; Africa 3, 4; HG12; HG16; Variant B18; TUB2; and atypical Guiana, spanning countries across South, Central, and North America; the Caribbean; and Africa [77]. Conventional genotype designations also include Type I, Type 1; Type II, Type 2 (clonal); Type II, Type 2 (variant); Type III, Type 3; Type 12, atypical, exotic (including Type X and Type A); Type BrI, atypical, exotic, Africa 1; Type BrII, atypical, exotic; Type BrIII, atypical, exotic; Type BrIV, atypical, exotic; and Chinese 1, atypical, exotic [90]. This layered genetic architecture, sustained across geographically distinct lineages rather than collapsing into a single dominant clone, gives the parasite a broad reservoir of phenotypic variation from which differences in virulence and host response can emerge.

That genetic diversity is matched by a comparably flexible metabolic architecture. Bioinformatic, genetic, and biochemical studies record that *T. gondii* expresses three fatty acid synthetic pathways localized to different cellular compartments: a cytosolic FASI‐like pathway producing palmitic acid; a FASII present in the apicoplast producing myristic and palmitic acids, in addition to lipoic acid; and an elaborate fatty acid elongation pathway compartmentalized in the ER and responsible for producing very long‐chain monounsaturated fatty acids [7, 91, 92]. Within the FASI pathway, gene structures reveal a single large polypeptide harboring the *ACP*, *FabD*, *FabH*, *FabG*, *FabZ*, and *FabI* activities. This pathway appears to primarily supply bulk products, mainly palmitic acid, to the apicoplast FASII, indicating that the FASI and FASII pathways act in concert to satisfy the parasite′s fatty acid requirements. Acetyl‐CoA is carboxylated to malonyl‐CoA by an acetyl coenzyme A carboxylase (ACC) using bicarbonate as the carboxyl source, biotin as a cofactor, and ATP as the energy source [93].

*FABZ* participates in fatty acid biosynthesis by catalyzing the dehydration of *β*‐hydroxyacyl‐acyl carrier protein dehydratase (*TgFABZ*) to trans‐2‐acyl‐ACP, making it a key enzyme in both fatty acid biosynthesis and the dehydration step of fatty acid synthesis [94]. RT‐PCR analysis confirms that *TgFABZ* is expressed in both tachyzoites and bradyzoites, with the TgFABZ protein localized to the apicoplast, an organelle unique to *T. gondii* [95, 96]. A report on nuclear‐encoded proteins of apicomplexan parasites, covering ribosomal protein genes (*rps9* or *S9* and *rpl28* or *L28*) and fatty acid biosynthesis genes (*acpP*, *fabH*, and *fabZ*, encoding ACP, *β*‐ketoacyl‐ACP synthase III, and *β*‐hydroxyacyl‐ACP dehydratase, respectively), demonstrated the localization of S9 and ACP to the apicoplast of *T. gondii* tachyzoites [7].

Two well‐known representative species within the Apicomplexa phylum, *T. gondii* and *Plasmodium* spp., encode a complete heme biosynthesis pathway in their genomes [6]. Eight heme biosynthetic enzymes within this pathway are distributed across three subcellular locations: the mitochondrion, cytoplasm, and apicoplast [6, 97, 98]. Heme typically serves as a prosthetic group in proteins such as cytochromes, which play an essential role in mitochondrial respiration through the electron transport chain in parasites [99, 100]. Toxoplasma also encodes orthologs of two other hemoproteins, cytochrome P450 and catalase, suggesting heme is likely involved in its detoxification processes [6]. A separate route, the mevalonate (MVA) pathway, begins with two molecules of acetyl‐CoA and requires six enzymes for isopentenyl pyrophosphate (IPP) synthesis, whereas the 2C‐methyl‐D‐erythritol 4‐phosphate (MEP) pathway uses seven enzymes to convert D‐glyceraldehyde‐3‐phosphate (GA3P) and pyruvate to IPP [101].

*T. gondii* also maintains a functional pentose phosphate pathway (PPP), comprising several enzymes distributed across the cytoplasm and nucleus [102, 103]. The PPP includes oxidative and nonoxidative branches, the latter consisting of potentially reversible steps. Glucose enters the oxidative PPP through reactions catalyzed by glucose‐6‐phosphate dehydrogenase and 6‐phosphogluconate dehydrogenase (6PGDH), generating ribulose‐5‐phosphate (Ru5P) and other glycolytic metabolites that are eventually converted into ribose‐5‐phosphate (R5P) through the nonoxidative PPP. This product, R5P, is vital for nucleotide synthesis and cell growth and survival [104, 105]. The nonoxidative PPP itself relies on transketolase (TKT) activity, which catalyzes two reversible reactions: the interconversion of R5P and xylulose‐5‐phosphate (Xu5P) to and from sedoheptulose‐7‐phosphate (S7P) and GA3P, and the conversion of erythrose‐4‐phosphate (E4P) and Xu5P to and from fructose‐6‐phosphate (F6P) and GA3P [106].

Alongside these metabolic pathways, *T. gondii* deploys molecular machinery that actively shapes the host immune response. The NF‐*κ*B pathway, composed of homodimers or heterodimers containing p65, p50, p52, c‐Rel, and RelB subunits, plays a pivotal role in regulating the expression of various inflammatory genes [107, 108], and its activity is modulated depending on which *T. gondii* genotype is present. *GRA15* from Type II parasites, for example, positively activates the NF‐*κ*B pathway in both mouse and human cells [109], whereas Type I *GRA7* initiates mitogen‐activated protein kinase (MAPK) and NF‐*κ*B signaling through production of NADPH oxidase‐dependent reactive oxygen species and Myd88‐dependent TRAF6 activation [110, 111]. Toxoplasma *GRA7* also induces a strong antibody response, making it a potential vaccine candidate and a promising serodiagnostic marker for toxoplasmosis [90, 112, 113].

*TgMIF* is a biochemically active protein capable of inducing IL‐8 production from human peripheral blood mononuclear cells (PBMCs) while also activating MAPK/ERK pathways in murine bone marrow–derived macrophages. *T. gondii* encodes a single copy of *MIF*, expressed in both tachyzoite and bradyzoite forms, and its presence may facilitate parasitism across definitive (Felidae), intermediate (*Mus musculus*), and incidental (*Homo sapiens*) hosts [114]. Alignment of TgMIF from three strains in one study showed 99.7% nucleotide sequence identity and 100% amino acid identity, while interspecies sequence alignments revealed 59% amino acid sequence similarity and 26% identity with mammalian host MIFs and 41.7% similarity with other parasite MIFs [115]. This tachyzoite–bradyzoite duality extends to storage metabolism: Bradyzoites accumulate abundant amylopectin granules and numerous micronemes, in contrast to tachyzoites, which lack amylopectin but carry fewer micronemes. Only UDP‐glucose pyrophosphorylase and UDP‐glucose‐utilizing amylopectin synthase have been identified in *T. gondii* for this purpose [116]. Transcripts for catabolic enzymes, including the *R1* protein, *α*‐glucan phosphorylase, *α*‐glucosidase, and *α*‐amylase, are preferentially expressed in bradyzoites, whereas enzymes involved in amylopectin synthesis, such as glycogenin, amylopectin synthase, and branching enzyme, are preferentially expressed in tachyzoites, though detectable at lower levels in bradyzoites as well [117].

This tachyzoite–bradyzoite interconversion is itself a direct response to host immune pressure. After an infected host detects the presence of *T. gondii* and activates its immune system, tachyzoites transform into bradyzoites and form cysts within the central nervous system or muscle tissue. The tissue cyst is a membranous structure containing lectins such as N‐acetylglucosamine and N‐acetylgalactosamine, which allow *T. gondii* to persist without triggering the host immune response [118]. *T. gondii* is additionally able to recognize, bind, and penetrate virtually any mammalian cell type using a repertoire of proteins released from late secretory organelles and a distinctive form of gliding motility, or glideosome, that depends critically on actin filaments and myosin. Comprehensive proteomic and glycomic analyses show that several key components of host cell–*T. gondii* interactions are N‐glycosylated [119, 120], carrying oligomannosidic (Man(5‐8)(GlcNAc)2) and paucimannosidic (Man(3‐4)(GlcNAc)2) sugars that are rarely present on mature eukaryotic glycoproteins [119].

The parasite′s membrane composition further reflects this adaptive specialization. Quantification of the phospholipid profile of *Toxoplasma* revealed that phosphatidylcholine is the most prevalent lipid, accounting for about 75% of total phospholipids, followed by phosphatidylethanolamine (10%), phosphatidylinositol (7.5%), phosphatidylserine (6%), and phosphatidic acid (1%) [121]. The parasite also contains relatively high levels of ceramide phosphoethanolamine with a fatty amide profile restricted to 16‐ and 18‐carbon species and carries greater amounts of shorter‐chain fatty acids in its polar lipids compared to its hosts [91]. Diacyl phosphatidylcholine with two saturated acyl chains of 12, 14, or 16 carbons makes up over 11% of the parasite′s phosphatidylcholine compared to less than 3% of host phosphatidylcholine molecular species [122].

Finally, *T. gondii* membranes contain farnesylated and geranylgeranylated proteins [123, 124]. Two farnesyl‐diphosphate synthase homologues identified in *Toxoplasma* localize to the mitochondria; this bifunctional enzyme catalyzes the formation of both farnesyl diphosphate and geranylgeranyl diphosphate [124]. The tachyzoite form of *T. gondii* also expresses a novel nucleoside triphosphate hydrolase (NTPase), first described in 1983 [125], which constitutes approximately 8% of total tachyzoite protein [125, 126].

Taken together, these molecular features are not isolated biochemical curiosities but the direct basis of the parasite′s success as a global pathogen. The genetic diversity spanning clonal, atypical, exotic, and recombinant lineages provides the raw variation from which differences in virulence and clinical presentation arise across regions and host populations. The parasite′s layered metabolic flexibility, spanning parallel fatty acid synthesis routes, a complete heme biosynthesis pathway, dual isoprenoid precursor pathways, and a functional PPP, allows it to sustain growth and replication across the metabolically distinct environments of its many intermediate and definitive hosts. Its capacity for rapid tachyzoite–bradyzoite interconversion, paired with immune‐modulating effectors such as *GRA15*, *GRA7*, and *TgMIF*, allows it to shift between aggressive proliferation and immune‐evading dormancy in direct response to host defenses, sustaining chronic infection while limiting the host′s ability to clear it. Together, these adaptations explain why *T. gondii* has achieved such an extensive host range and worldwide distribution and why the severity of infection varies so markedly between individuals and populations. Future work that maps specific genotype–metabolism–virulence relationships, particularly in underrepresented atypical and African lineages, will be essential for translating this molecular understanding into genotype‐informed diagnostics and more precisely targeted therapeutic strategies.

## 6. Epidemiological Implications of Toxoplasmosis

The biological and geographical prevalence of *Toxoplasma* infection ranges from less than 1% in young adults to 90% in older adults. An estimated 30%–65% of all persons worldwide are infected with *Toxoplasma*. Serological studies indicate that pregnant women in Brazil have a prevalence rate of 47.9%, whereas dairy goats in Shaanxi Province, Northwestern China, have a seroprevalence of 32.34% [127]. Sanitation levels, contact with soil, exposure to raw meat, and handling slaughtered animal blood are all general risk factors for *T. gondii* infection among people who own domestic animals [128]. More importantly, people with poor sanitation have a higher infection rate. The frequency of *T. gondii* varies between areas of the United States, though this depends on differences in temperature, cat numbers, and human activity [129]. Most of the evidence about infections originates from animal prevalence studies and epidemiological research. Transmission by meat is important in the United States, as indicated by the spike of illnesses in teenagers and the low prevalence in early children [130]. Clinically, toxoplasmosis affects just a tiny fraction of exposed adult humans or animals [131]. However, whether the severity of toxoplasmosis in immunocompetent people results from the parasite strain, host diversity, or another cause is unknown. *Toxoplasma* infections caused by oocysts are significantly more severe than those caused by tissue cysts and bradyzoites through the natural oral route, regardless of the dose [132].

Among different regions, seroprevalence elevation is in women from Latin America, northern Africa, Eastern Europe, sub‐Saharan Africa, and Western Europe [133]. With increasing age, certain regions in the Netherlands, relatively low education levels, cat ownership, gardening, and consumption of raw meat, especially raw/undercooked pork, were some of the identified risk factors for acute toxoplasmosis in the Netherlands [134]. Additionally, the seroprevalence of *T. gondii* infection varies significantly across different populations and regions, including children, humans, and animals in Africa [135]. A study conducted among 400 participants in Africa identified the seroprevalence of IgG and IgM/IgG *T. gondii* antibodies at 57.3% and 57.8%, respectively, and found that raw vegetables were ingested by 84.3% of the participants. Again, 36.2% of the participants ate raw meat without cooking, 42.5% had constant contact with soil, and 20.5% owned cats [136]. Furthermore, socioeconomic status is a significant factor influencing the prevalence of *Toxoplasma* infection [135]. A cross‐sectional study conducted from February 2006 to June 2010 in Elche, Spain, assessed the seroprevalence of *T. gondii* among pregnant women. Adherence to screening was high in both groups: 91.9% among migrants and 92.2% among Spaniards. The seroprevalence of IgG anti‐*T. gondii* antibodies was significantly higher in migrant women (41.4%) compared to Spaniards (12.0%), with an odds ratio (OR) of 5.2. Notably, no Asian pregnant woman tested positive. Seroprevalence also increased with age among migrants: 38.2% in women aged 15–25 years, 40.7% in those aged 26–35 years, and 52.8% in those aged 36–45 years. These findings highlight a higher *T. gondii* infection rate in migrant pregnant women compared to the native population, emphasizing the need for targeted prevention, especially for women from Asia [133]. In a northern Mexican city, the prevalence of *T. gondii* infection was 12.1% in men and 17.5% in women, with research identifying consuming pigeon and iguana meat, as well as contact with soil and neighborhood cats, as risk factors for infection in women [137]. In Europe, particularly Germany, *T. gondii* infections are predominantly acquired through the consumption of raw pork sausage products. The seroprevalence of IgG antibodies against *T. gondii* is nearly 50% among adults aged 18–79, increasing from 20% in young adults (18–29 years) to 77% in those aged 70–79. Higher seropositivity rates are observed in men, cat owners, and individuals with a BMI ≥ 30. Conversely, vegetarian diets and high socioeconomic status are associated with lower infection rates [138]. A 10‐year study conducted in Tanzania also gave some interesting results. An average of 19 hospital deaths per year from 2006 to 2015 accounted for 0.08% of total hospital deaths. Adults were predominantly affected, with equal distribution between males and females, although global studies often find higher rates in women, especially those of reproductive age. A key risk factor is the strong association with HIV, as 75% of toxoplasmosis deaths occur in HIV‐positive individuals, highlighting the vulnerability of immunosuppressed populations. Cerebral toxoplasmosis is particularly deadly, making up 15.4% of these deaths and posing a major threat to AIDS patients. Geographically, higher mortality rates are seen in the southwestern highlands, southern, and eastern regions, especially in Mbeya and Lindi. In Mbeya, this is linked to high HIV prevalence, whereas in Lindi, local practices such as rodent hunting and consumption likely contribute to the risk [139]. Some parts of Taiwan also found an increasing trend in the incidence of toxoplasmosis, with the incidence rate highest in young male patients, during summer, and in the eastern region [140]. As identified by most researchers, increasing age; consumption of raw oysters, mussels, and clams; and owning a cat were significant risk factors for toxoplasmosis in the United States. Being pregnant or a woman of childbearing age was also associated with an increased risk [141].

In Poland, a study found no link between *T. gondii* DNA in the brain and excessive alcohol consumption [142], while another Polish study found higher levels of anti‐*T. gondii* antibodies in people who tested positive for blood alcohol [143]. In Finland, researchers did not find an association between *T. gondii* infection and alcohol use disorders [144]. However, in patients with heart disease, those who consumed alcohol had a higher rate of *T. gondii* seropositivity. Overall, the connection between *T. gondii* and alcohol use is inconsistent across different studies [145].

Toxoplasmosis has been linked to an increased risk of various mental and physical health disorders. Mental health disorders, particularly schizophrenia, are linked to toxoplasmosis. A longitudinal study confirmed the causal role of toxoplasmosis in the development of schizophrenia. *Toxoplasma*‐seropositive schizophrenia patients exhibited typical changes in brain morphology, such as gray matter reduction. They expressed more prominent positive symptoms of schizophrenia and had a 15‐fold higher probability of having a continuous course of disease than *Toxoplasma*‐free patients. However, fewer studies have shown the association of toxoplasmosis with other mental health disorders, such as bipolar disorder, obsessive–compulsive disorder, learning disorder, autism, and anxiety disorder.

Food safety remains a significant global public health challenge, particularly due to zoonotic pathogens that can spread from animals to humans. *T. gondii* infects diverse livestock species, serving as both reservoirs for zoonotic transmission and subjects of agricultural burden [8]. Vulnerable populations such as pregnant women face severe consequences and life‐threatening complications when infected [146]. Although there has been great advancement in food safety systems, *T. gondii* still remains a public health concern due to evolving farming practices as well as consumers′ preferences for undercooked meats. Dietary practices vary across countries, thereby increasing the infection rate in livestock. For instance, people in France and Norway have strong appetites for undercooked lamb, which exposes them to *T. gondii* infection from sheep. However, in Poland, undercooked pork is the greatest risk, thereby confirming the different dietary practices [147]. Generally, about 60% of *T. gondii* infections can be found in farms with free‐ranging pigs without controlled conditions allowing outdoor access. Pigs reared in confinement and controlled management conditions have less than 1% *T. gondii* infections due to their inaccessibility to cats and rodents [148]. A global distribution of toxoplasmosis in humans is summarized in Figure 2.

**Figure 2:**
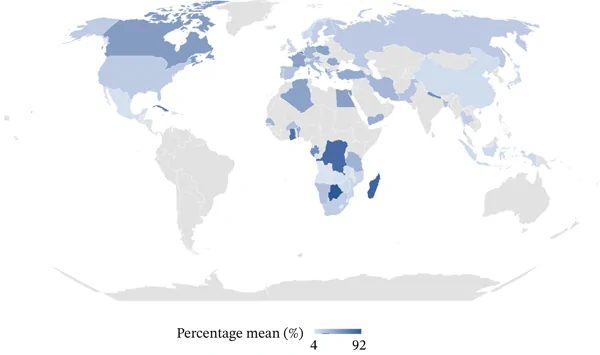
Global distribution of toxoplasmosis in humans. The figure shows the minimum (4.0%) and the maximum (92.0%) mean prevalence of toxoplasmosis across the various regions. The average prevalence for each endemic region has been indicated in brackets: Africa (38.5%) [149, 150], Asia (21.23%), North America (39.63%), Europe (28.7%) [150], and Middle East (33.3%) [151, 152].

## 7. Economic Implications of Toxoplasmosis

The economic impact of toxoplasmosis manifests through various channels, including healthcare costs, productivity losses, and impacts on agricultural sectors as well as tourism and wildlife (Table 1) [132]. Addressing the economic dimensions of toxoplasmosis would thus require a multifaceted approach, encompassing improved healthcare infrastructure, targeted interventions in high‐risk populations, and enhanced awareness to mitigate its socioeconomic impact.

**Table 1:** Economic impact of toxoplasmosis in selected countries.

Country	Sector affected	Estimated economic impact	Major consequences	Reference
United States	Human health (congenital toxoplasmosis)	> $1 billion annually	Medical treatment, lifelong healthcare, productivity losses, and special education	Jones et al. [132]
Brazil	Sheep production	Substantial but not nationally quantified	Abortion storms, reduced fertility, and decreased productivity	Dubey et al. [153]
Uruguay	Sheep production	$1.4–4.68 million annually	Reproductive losses, abortions, and reduced flock productivity	Freyre et al. [154]
United Kingdom (Great Britain)	Sheep production	Output losses: $15.4–23 million/year; treatment: ~$1.3 million/year	Abortions, stillbirths, and disease control costs	Bennett et al. [155] and Bennett and Ijpelaar [156]
East African countries (Kenya, Tanzania, and Uganda)	Wildlife conservation and tourism	Not quantified	Wildlife mortality, biodiversity loss, and reduced ecotourism revenue	Bokaba et al. [157]
France	Human health	Estimated several hundred million euros annually	Congenital toxoplasmosis management, prenatal screening, treatment, and long‐term disability costs	Torgerson and Mastroiacovo [20]
Netherlands	Human health	Approximately €45–50 ($51.96–57.73) million annually	Healthcare costs and productivity losses associated with congenital and acquired toxoplasmosis	Havelaar et al. [158]
Australia	Sheep industry	Tens of millions of Australian dollars annually	Lamb mortality, abortions, and reduced reproductive performance	Legge et al. [159]
New Zealand	Sheep industry	Several million NZ dollars annually	Reproductive failure, reduced lamb production, and veterinary costs	Dempster et al. [160]

Toxoplasmosis contributes significantly to global healthcare expenditure. Treatment costs, diagnostic procedures, and management of complications impose a substantial financial burden on healthcare systems. In the United States alone, the annual economic burden of CT has been estimated to exceed $1 billion, encompassing medical care, productivity losses, and special education services [132].

The agricultural sector is not immune to the economic repercussions of toxoplasmosis. Infected animals, particularly livestock, suffer reproductive losses, decreased meat and milk production, and increased mortality rates [161, 162]. For instance, in Brazil, toxoplasmosis in sheep leads to substantial economic losses due to abortion storms and reduced fertility rates, affecting both smallholder farmers and large‐scale enterprises [153]. Annual losses with toxoplasmosis in sheep flocks in Uruguay ranged from 1.4 to 4.68 million dollars [154].

Bennett et al. published several studies on the direct costs associated with endemic diseases of livestock in Great Britain, including toxoplasmosis in sheep [155]. In one such study, annual costs of 12–18 million pounds (15.4–23 million dollars equivalent) for output losses (L) and almost 1 million pounds (1.3 million dollars) for treatment (T), but no costs for prevention (P), were estimated. An incidence of ovine toxoplasmosis in ewes of 1.2% and 2.2% was assumed. In an update of this study, the authors estimated that about 334,000 (range: 136,000–526,000) sheep were affected per year, with disease effects of 9.1 (range: 3.7–14.1) million pounds (due to abortion or stillbirth) and 3.2 (range: 2.7–5.6) million pounds for disease control, which translates into 4.7–18 million dollars and 3.5–7.2 million dollars, respectively. It was assumed that toxoplasmosis caused 50,000 (range: 8000–116,000) severely affected sheep in Great Britain per year [155, 156].

Toxoplasmosis can also impact tourism and wildlife conservation efforts. In regions where wildlife tourism is a significant economic driver, such as East Africa, the disease′s prevalence in wildlife poses a threat to endangered species and ecotourism revenues. Studies have highlighted the potential economic losses associated with toxoplasmosis‐related wildlife mortality and reduced tourist visitation to affected areas [157].

In occupational settings, toxoplasmosis can lead to productivity losses and occupational hazards, especially in occupations involving contact with soil or animals. For instance, in agricultural communities in Europe, where soil contamination with *T. gondii* oocysts is prevalent, toxoplasmosis poses occupational health risks to farmers, veterinarians, and abattoir workers, contributing to absenteeism and reduced productivity [163].

Beyond direct economic costs, toxoplasmosis can exacerbate socioeconomic disparities and impact vulnerable populations disproportionately. Limited access to healthcare services, inadequate sanitation infrastructure, and lack of awareness contribute to higher disease prevalence rates in low‐income communities. In regions like sub‐Saharan Africa, where toxoplasmosis prevalence is high, the disease can perpetuate cycles of poverty by affecting cognitive development in children and reducing workforce productivity in adulthood [164].

## 8. Drug and Vaccine Development

The search for new drugs and therapeutic regimens for toxoplasmosis is ongoing, with a focus on reducing adverse effects and improving efficacy [165]. Recent progress in drug discovery has led to the identification of novel compounds, although some challenges remain in eradicating tissue cysts and developing effective and best treatments for pregnant women [166]. Also, the current drugs target the acute form of the disease with limited effect on the chronic form, though promising late‐stage drug candidates are being explored [29].

The current first‐line treatment for toxoplasmosis includes a pyrimethamine‐based regimen, mainly comprising three drugs: pyrimethamine, folinic acid, and sulfadiazine [167]. The pyrimethamine drug targets an enzyme involved in folic acid synthesis, specifically dihydrofolate reductase, responsible for the reduction of dihydrofolic acid to tetrahydrofolic acid. This blocks the synthesis of purines and pyrimidines needed for DNA synthesis [168]. The inhibitory activity of the drug on the parasite′s folic acid synthesis is enhanced when used in combination with sulfadiazine. Sulfadiazine competitively inhibits another enzyme in the pathway called dihydropteroate synthetase. It is, however, not advisable to take this combination during the first trimester of pregnancy, as its dosage is known to cause abnormalities in the physiological development of the fetus [168]. To prevent the possible teratogenicity and myelosuppression, the combined dose is complemented with folinic acid, which is the active metabolite of folic acid [169, 170]. In cases where patients are allergic to sulfur drugs, a clindamycin‐containing regimen is given [171].

Clindamycin has been found to target and interfere with the replication of *T. gondii* within parasitophorous vacuoles [172]. However, it is important to note that in some cases of toxoplasmosis, clindamycin administration has been associated with paradoxical effects, leading to disseminated toxoplasmosis and manifestations like hepatitis and interstitial pneumonia [167, 173]. Pyrimethamine–clindamycin, along with steroids, has shown promise in improving the prognosis of cerebral toxoplasmosis with severe edema [174, 175]. The World Health Organization recommends combining clindamycin with other drugs such as quinine, artesunate, or pyrimethamine for the treatment of toxoplasmosis, particularly in pregnant women [176]. Clindamycin has also been utilized in intravitreal therapy for ocular toxoplasmosis, providing an alternative treatment option for patient′s intolerant or resistant to systemic therapies [177, 178]. Clindamycin is, however, less effective compared to the conventional treatment regimen.

In place of a pyrimethamine‐based regimen, co‐trimoxazole (a combination of trimethoprim and sulfamethoxazole) can be used as an alternative. It is indicated as the first‐line drug regimen for both curative treatment and prophylaxis of toxoplasmic encephalitis, with a high success rate (85.5%) in patients responding to the drug treatment [179]. Its mode of action also involves the inhibition of *T. gondii* replication within the host cells [180]. Studies have also shown its effectiveness in the treatment of CT, making it a recommended prophylaxis for both *Pneumocystis* pneumonia and toxoplasmosis in HIV‐infected patients [181, 182].

Another drug alternative for the treatment of toxoplasmosis is azithromycin. Azithromycin′s mode of action involves inhibiting protein synthesis of *T. gondii* in the tachyzoite and bradyzoite stages [183]. Azithromycin can be employed in cases of ocular toxoplasmosis, where it can be an effective alternative for patients who cannot tolerate standard therapies [184]. When used in combination with sulfadiazine, it has demonstrated a synergistic effect in the treatment of acute toxoplasmosis in mice [185]. This is also seen in the treatment of cerebral toxoplasmosis in an AIDS patient, where the combination of azithromycin and pyrimethamine led to successful treatment [186].

Spiramycin is a macrolide antibiotic that is sometimes given to pregnant women to prevent complications in the uterus after birth. Although it does not easily cross the placental barrier, it accumulates in the placenta and helps prevent the transmission of *T. gondii* from mother to baby [169]. However, if fetal or neonatal toxoplasmosis is diagnosed, spiramycin treatment is stopped, and conventional treatment with antiparasitic medications is started [187, 188]. There are several challenges associated with the use of these drugs to treat toxoplasmosis. One issue is drug resistance, which leads to therapeutic failure in around 10% of patients during initial therapy and 10%–20% of individuals who experience relapse during suppressive therapy [171]. Table 2 depicts the chemical structures of the common drugs used in the treatment of toxoplasmosis.

**Table 2:** Chemical structures of common anti‐*T. gondii* drugs.

Structure	Name	Reference
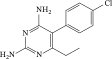	5‐(4‐Chlorophenyl)‐6‐ethylpyrimidine‐2,4‐diamine (pyrimethamine)	Dunay et al. [167]
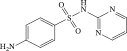	4‐Amino‐N‐pyrimidin‐2‐ylbenzenesulfonamide (sulfadiazine)	Dunay et al. [167]
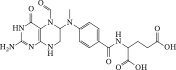	2‐[[4‐[(2‐Amino‐5‐formyl‐4‐oxo‐3,6,7,8‐tetrahydropteridin‐6‐yl)methylamino]benzoyl]amino]pentanedioic acid (folinic acid)	Dunay et al. [167]
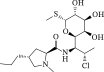	(2S,4R)‐N‐[(1S,2S)‐2‐Chloro‐1‐[(2R,3R,4S,5R,6R)‐3,4,5‐trihydroxy‐6‐methylsulfanyloxan‐2‐yl]propyl]‐1‐methyl‐4‐propylpyrrolidine‐2 carboxamide (clindamycin)	Vuković et al. [172]
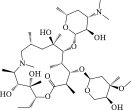	(2R,3S,4R,5R,8R,10R,11R,12S,13S,14R)‐11‐[(2S,3R,4S,6R)‐4‐(Dimethylamino)‐3‐hydroxy‐6‐methyloxan‐2‐yl]oxy‐2‐ethyl‐3,4,10‐trihydroxy‐13‐[(2R,4R,5S,6S)‐5‐hydroxy‐4‐methoxy‐4,6‐dimethyloxan‐2‐yl]oxy‐3,5,6,8,10,12,14‐heptamethyl‐1‐oxa‐6‐azacyclopentadecan‐15‐one (azithromycin)	Değerli et al. [183]
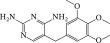	5‐[(3,4,5‐Trimethoxyphenyl)methyl]pyrimidine‐2,4‐diamine (trimethoprim)	Beraud et al. [179]
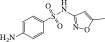	4‐Amino‐N‐(5‐methyl‐1,2‐oxazol‐3‐yl)benzenesulfonamide (sulfamethoxazole)	Beraud et al. [179]
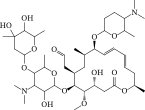	2‐[(4R,5S,6S,7R,9R,10R,11E,13E,16R)‐6‐[5‐(4,5‐Dihydroxy‐4,6‐dimethyloxan‐2‐yl)oxy‐4‐(dimethylamino)‐3‐hydroxy‐6‐methyloxan‐2‐yl]oxy‐10‐[5‐(dimethylamino)‐6‐methyloxan‐2‐yl]oxy‐4‐hydroxy‐5‐methoxy‐9,16‐dimethyl‐2‐oxo‐1‐oxacyclohexadeca‐11,13‐dien‐7‐yl]acetaldehyde (spiramycin)	Castro‐Filice et al. [169]

Various studies have been conducted with continuous effort to develop the best and most effective vaccine against this parasite, though no effective vaccine for toxoplasmosis in humans currently exists [189]. Research has shown that single or a few antigens may only provide partial protection against toxoplasmosis [190]. Ovilis Toxovax, a live‐attenuated vaccine based on the tachyzoites of *T. gondii* S48 strain, is currently the only commercially available toxoplasmosis vaccine that is specifically designed to reduce fetal abortion in healthy sheep. However, it has several limitations, such as its extremely short half‐life of 10 days, and is practically impossible to administer in humans due to ethical reasons and safety concerns. Also, the toxovax vaccine is commercially marketed in the United Kingdom, New Zealand, France, and Ireland [191], with vaccination inducing immunity that protects against abortion for at least 18 months after the initial vaccination ([192–194]. It is not considered an appropriate vaccine for use in humans also due to its live‐attenuated form having the potential to revert to a pathogenic form [195].

Studies have delved into the usage of adjuvants such as chitosan nanospheres to enhance the immunogenicity of *Toxoplasma* lysate vaccines in mice [196]. Furthermore, the evaluation of different immunization regimens with adjuvanted *T. gondii* vaccines is still ongoing [197]. Live‐attenuated strains like ME49*Δ*cdpk3 have demonstrated effectiveness in inducing immune responses against acute and chronic toxoplasmosis, making them potential vaccine candidates [198]. Novel approaches such as lactate fermentation mutants lacking specific enzymes have also shown promise [199].

DNA vaccines/subunit vaccines as well as nanoparticle‐based vaccines have been developed and tested for their efficacy and potential in inducing immune responses against *T. gondii* [200, 201].

Recombinant proteins such as phosphoglycerate mutase 2 have also been investigated for their potential in conferring protective immunity against toxoplasmosis [202]. These novel approaches have shown promise in rodent studies, but translating these results to clinical studies remains a challenge [203]. Recombinant proteins derived from *T. gondii* have been identified as promising candidates for vaccine development. These proteins, such as Surface Antigen 1 (SAG1), SAG2, Rhoptry Protein 2 (ROP2), and microneme proteins, play crucial roles in the parasite′s life cycle and immune‐evasion strategies [204]. For instance, Pinzan et al. demonstrated that vaccination with recombinant microneme proteins could elicit a Th1 immune response, which is essential for conferring protection against *T. gondii* infection in experimental models [204]. This aligns with the findings of Naeem et al., who reported that intranasal immunization with SAG1 polymeric nanospheres induced a robust Th1‐oriented humoral response, further supporting the potential of recombinant proteins in vaccine formulations [59]. Grzybowski et al. [205] also used recombinant ROP5 and ROP18 proteins, which were expressed and purified from *E. coli*. The proteins were administered to mice with poly I:C as an adjuvant, and the immune response was evaluated through antibody and cellular assays. The immunization generated both cellular and humoral specific immune responses, with some predominance of IgG1 antibodies, and led to partial protection against acute and chronic toxoplasmosis, inducing a higher immune response compared to single‐antigen vaccines. Chyb et al. [206] experimented with recombinant chimeric proteins SAG2‐GRA1‐ROP1 and SAG1‐MIC1‐MAG1‐GRA2, and the results indicated long‐lasting immunity against *T. gondii*, making them attractive candidates for further research aimed at boosting their immunogenicity and immunoprotective capacity. The use of multiepitope constructs has also been a focal point in the research. These constructs can present multiple epitopes simultaneously, enhancing the immune response and providing broader protection against various strains of *T. gondii* [197].

Moreover, the design of delivery systems such as nano‐ and microparticle formulations continues to be vital in the development of effective *T. gondii* vaccines. These systems have the potential to improve the stability and absorption of recombinant proteins, hence improving immunogenicity [207, 208]. For example, the use of PLGA microspheres to deliver recombinant SAG1 and GRA2 has shown promising results, as they elicited significant immune protection against *T. gondii* infections [207]. However, challenges persist in translating findings from mouse models to other animal species, primarily due to the reliance on specific adjuvants such as complete or incomplete Freund′s adjuvant, which complicates the correlation of efficacy across different platforms [209]. The ethical considerations surrounding vaccine trials across varied species also remain a hurdle that requires further scholarly attention.

## 9. Functional Significance of Medicinal Plants for Toxoplasmosis

The persistence of toxoplasmosis as an economic burden is inseparable from the limitations of its frontline pharmacological treatment. Pyrimethamine‐based regimens, still the standard of care in most settings, require prolonged administration, carry significant hematological toxicity, and demand regular clinical monitoring that is costly to sustain over months of therapy, particularly for congenital and ocular disease, where treatment courses extend well beyond acute infection. In parallel, livestock losses linked to *T. gondii* infection continue to drain agricultural income in regions where veterinary pharmaceuticals are expensive or difficult to access. Plant‐based therapeutics offer a plausible route around both problems: Many of the botanicals investigated to date are inexpensive to cultivate or source locally, require minimal processing, and, in several cases, have shown efficacy comparable to conventional drugs without the same toxicity profile, positioning them as scalable, low‐cost interventions rather than niche alternatives.

*Artemisia annua* L., from which artemisinin and related compounds are derived, may inhibit multiple steps of the *T. gondii* lytic cycle. It is known for its antimalarial properties and has been investigated for its potential against other parasites, including *T. gondii*, despite reported resistance among some strains [19, 210]. Synthetic unsaturated carba derivatives of artemisinin serve as therapeutic agents for preventing and treating toxoplasmosis in humans [211]. Five of seven new artemisinin derivatives effectively inhibit *T. gondii* growth, with three being relatively noncytotoxic [212]. The same five derivatives inhibited tachyzoite replication, as well as host cell attachment and invasion, to the same or greater extent than the parent compound artemisinin [213]. Artemisinin derivatives disrupt the parasite′s calcium homeostasis, alter mitochondrial function, and induce toxic reactive oxygen species, targeting multiple stages of the parasite′s lytic cycle [212].

Allicin, a compound found in *Allium sativum* L. (garlic), exhibits antimicrobial properties [214], and other reports indicate potential efficacy against *T. gondii* [215]. Alnomasy [215] found that the essential oil of *A. sativum* demonstrated significant anti‐*Toxoplasma* activity against *T. gondii* tachyzoites at various concentrations, with the highest efficacy at 150 *μ*g/mL. Pretreatment of infected mice with *A. sativum* essential oil at doses of 200, 400, and 600 *μ*g/kg/day reduced tachyzoite numbers and mortality rates. The study also showed that *A. sativum* essential oil has prophylactic effects that can promote the innate immune system and inhibit hepatic injury in mice with acute toxoplasmosis [214]. Overall, allicin inactivates *T. gondii*′s essential enzymes by binding to their thiol (‐SH) groups, directly killing tachyzoites and blocking motility [216].

*Azadirachta indica* A. Juss. (neem) extracts have been investigated for their potential anti‐*Toxoplasma* activity, with studies indicating that neem compounds may inhibit parasite proliferation and eliminate intracellular development [217]. An investigation found that neem extract treatment improved toxoplasmosis outcomes in mice by increasing survival time and lowering tachyzoite load. Compared to septrin‐treated mice, histopathological examination revealed decreased lymphocyte infiltration and necrosis, as well as a significant increase in interferon gamma (IFN‐*γ*) levels [218]. Neem combined with *Cinnamomum camphora* (L.) also proved effective against virulent strains of *T. gondii* [219]. Neem extracts achieve this reduced tachyzoite load by blocking intracellular replication, boosting IFN‐*γ* production, and minimizing systemic tissue necrosis [220].

*Camellia sinensis* (L.) Kuntze (green tea) was tested on *T. gondii* strain trophozoites from infected mice in a study. Results showed that oolong and green tea had stronger protozoacidal effects than black tea [221]. The lowest effective concentration was 0.5%, and a half hour was not enough for the tea to completely kill the parasites. Distilled water was found to be more effective than phosphate buffer solution and normal saline in killing protozoa [221]. A follow‐up study indicated that treatments combining lincocin and green tea may be the best option to avoid the side effects of acute toxoplasmosis [222]. Green tea reflects a multitarget, multipathway mode of action driven mainly by catechins such as epigallocatechin 3‐gallate (EGCG), epigallocatechin (EGC), epicatechin‐3‐gallate (ECG), and epicatechin (EC). These compounds interact with host and parasite pathways involved in oxidative stress, membrane integrity, immune signaling, and inflammation, creating a synergistic antimicrobial effect [223]. Rather than relying on a single molecule, green tea′s diverse phytochemical profile collectively disrupts parasite viability, enhances host immune responses, and reduces inflammatory damage, consistent with its stronger protozoacidal activity relative to black tea and its potential to complement lincocin therapy in acute toxoplasmosis.

*Cinnamomum burmannii* (Nees and T. Nees) Blume methanol extract (cinnamon tea) is also effective against chronic *T. gondii* infection in mice [224]. Cinnamon aqueous extract is known to cause progressive disorganization of intracellular tachyzoites, resulting in their elimination without toxic effects to the host cell [217]. The extracts drive continuous physical and structural breakdown of tachyzoites directly within their protective vacuoles without harming host cells, and the methanol derivatives in particular show unique efficacy in clearing chronic‐phase tissue cysts [217].

Essential oil of *Curcuma longa* L. exhibited strong inhibitory effects on *T. gondii* tachyzoites. Curcumin, the active component in turmeric, showed anti‐*Toxoplasma* effects in vitro [225], improving the survival rate by the 9th day and decreasing the average number of peritoneal tachyzoites in infected mice [226]. It also interferes with parasite growth and replication, as demonstrated in mice when used in conjunction with nanoparticles [227]. *C. longa*′s antidepressant effects have been linked to modulation of oxidative stress and the cytokine network in the hippocampal regions of infected mice, reducing anxiety and depression symptoms and suggesting a role as an antidepressant against *T. gondii*‐induced affective disorders [225].

*Olea europaea* L. leaf extracts contain compounds like oleuropein that have demonstrated antiparasitic properties [228]. Oleuropein, isolated from *O. europaea*, was studied for its anti‐tachyzoite proliferation effect in mice, inhibiting cyst development in the liver and spleen similarly to sulfadiazine and pyrimethamine [229]. The same study showed good antigranuloma, antiapoptosis, and antinecrosis effects in *T. gondii*‐infected mice, with higher selectivity than sulfadiazine and pyrimethamine. Oleuropein inhibits *T. gondii* growth and intracellular replication and prevents the parasite′s entry into the cell [228], blocking initial attachment and physical entry of tachyzoites into host cells, halting intracellular multiplication, impeding tissue cyst formation, minimizing inflammatory granuloma development caused by parasite aggregation, and acting as an antiapoptotic and antinecrosis agent that protects host tissue from infection and cell death [229].

The hydroalcoholic extracts of *Lepidium perfoliatum* L. and *Lepidium sativum* L. seeds exhibited promising anti‐*Toxoplasma* activity through direct growth inhibition of *T. gondii*‐infected mouse cells [17, 18]. More broadly, the Brassicaceae family contains several medicinal plants with anti‐inflammatory, chemopreventive, insecticidal, antibacterial, antiviral, and antiparasitic effects, producing bioactive compounds such as glucosinolates that, upon hydrolysis, generate isothiocyanates and other sulfur‐containing compounds contributing to their therapeutic effects. These compounds penetrate infected cells to stop *T. gondii* expansion, disrupt the parasite′s development cycle, and initiate a downstream anti‐inflammatory cascade that modulates the host immune system to control infection‐provoked tissue inflammation while offering broad antimicrobial resistance [230]. As such, *Lepidium* species, alongside other Brassicaceae plants, hold potential as natural therapeutic agents against a variety of pathogens, including parasites [17, 18].

An in vitro study indicated that *Piper betle* L. extract was the most effective extract at inhibiting *T. gondii* growth in HFF cells among the Thai Piperaceae family tested. Treatment of experimental mice with *P. betle* extract for 7 days after infection with 1000 tachyzoites of the *T. gondii* PLK strain increased survival rates to 100% [231]. Methanol extraction of different fractions from *Pleopeltis crassinervata* T. Moore showed higher antiparasitic efficacy on RH strain tachyzoites than previous studies on *T. gondii* [231], with the highest toxoplasmicidal activity observed in the hexane fraction, which contained terpenes, had no antioxidant activity, and had the lowest extraction yield [231]. The pharmacology of *P. betle* L. extract centers on blocking *T. gondii* proliferation within host fibroblast cells, effectively neutralizing lethal parasitic challenges; when administered postinfection, its active compounds deliver a robust in vivo protective effect that raises host survival rates to 100% [232].

*Zingiber officinale* Roscoe extract has been shown to be effective against a variety of parasites, including *Schistosoma* and *Trypanosoma*. El‐Kady et al. [233] found that *Z. officinale* extract treatment significantly reduced cysts in *T. gondii*‐infected mouse brains, alleviated brain edema and inflammation, and reversed neuronal damage caused by the parasite. Its activity against *T. gondii* centers on a multiorgan system that halts parasite cyst development in the brain while providing broad multisystem cytoprotection, acting on a distinct neuroprotective axis to inhibit brain cell apoptosis, reverse neuronal degeneration, and resolve systemic inflammation across cerebral, hepatic, and pulmonary tissues (El kady et al., 2022). This suggests that ginger has neuroprotective properties that could be critical in preventing the long‐term neurological damage caused by toxoplasmosis. It also reduced inflammation in the liver and lungs, protected hepatocytes from infection‐induced degeneration, and inhibited apoptosis in the brain [234], suggesting the extract may be a safe and effective treatment for chronic toxoplasmosis.

Despite this accumulating preclinical evidence, and despite the long‐standing traditional use of various African botanicals for parasitic diseases, no completed human clinical trials have evaluated African medicinal plants for the treatment of *T. gondii* infection. Across multiple studies, extracts from African plants, including *C. longa* L., *Vernonia amygdalina*, *Elaeis guineensis*, *A. annua* L., *Vachellia tortilis*, *Z. officinale*, *L. perfoliatum* L., *L. sativum* L., and *Acacia nilotica*, have demonstrated tachyzoite growth inhibition, parasite load reduction, and immune‐modulating effects in laboratory settings, pointing to promising bioactive compounds such as phenolics, flavonoids, and norsesquiterpenoids with potential anti‐*Toxoplasma* activity [235]. Yet none of these extracts has progressed to human testing, and the gap is not simply a matter of insufficient research attention. Several structural barriers account for it. Many of the bioactive constituents identified in these plants, particularly polyphenols, terpenes, and glucosinolate‐derived isothiocyanates, suffer from poor oral bioavailability, being rapidly metabolized, poorly absorbed across the gut epithelium, or degraded before reaching systemic circulation at concentrations comparable to those effective in vitro or in rodent models. Dose–response and toxicity profiles established in mice also do not translate directly to humans, since interspecies differences in metabolism mean that a dose shown to be safe and effective in a mouse model cannot be assumed safe at the correspondingly scaled human dose without dedicated pharmacokinetic and toxicological studies, which remain largely absent for these compounds. Compounding this, whole‐plant extracts are chemically heterogeneous and vary with growing conditions, harvest timing, and extraction method, so standardization of active compound concentration between batches, let alone between different plant populations or geographic sources, is difficult to achieve and difficult to regulate, undermining the batch‐to‐batch consistency that clinical trial design and eventual drug approval require. Together, these bioavailability, toxicity, and standardization gaps mean that the plant extracts discussed here remain experimental leads for future drug development rather than clinical options, and closing that gap will require targeted investment in pharmacokinetic characterization, formulation strategies that improve absorption and stability, and standardized extraction protocols before African medicinal plants can move credibly from traditional and preclinical use toward regulated human therapeutics. Table 3 summarizes the mode of action of some plant species with reported anti‐*T. gondii* activities in vitro and in vivo.

**Table 3:** Plants with reported anti‐*T. gondii* activities.

Plant species	Family	Mode of action	Reference
*Artemisia annua* L.	Asteraceae	Decreases mortality rate and increases survival time; reduces parasite burden, affecting the viability and infectivity of tachyzoites, with shape distortions, peeling, erosions, and discontinuity observed both in vitro and in vivo.	El Zawawy [213]
*Artemisia Judaica* Lour.	Asteraceae	Strong anti‐*Toxoplasma gondii* activity, with an IC_50_ of 2.1 *μ*g/mL. Rich in terpenoids, flavonoids, and, in some species, artemisinin‐like sesquiterpene lactones, which are known to disrupt protozoan parasites by generating reactive oxygen species and interfering with mitochondrial function. Potent inhibition of tachyzoite growth and significant reduction in infection rate (6.7% vs. 29.1% untreated) suggest that its bioactive compounds impair parasite viability and invasion.	Abdou et al. [235]
*Allium sativum* L.	Amaryllidaceae	Promotes the innate immune system and proinflammatory cytokines and inhibits hepatic injury.	Alnomasy [215]
*Azadirachta indica* A. Juss	Meliaceae	Reduces tachyzoite count and viability; increases IFN‐*γ* expression, limiting parasite replication.	Rashed et al. [218]
*Berberis vulgaris* L.	Berberidaceae	Decreases mean number of tachyzoites as treatment progresses. Arrests parasite division and disrupts the lifecycle of the tachyzoites while regulating host‐immune, inflammatory, and oxidative pathways.	Mahmoudvand et al. [236]
*Carmellia sinensis* (L.) Kuntze	Theaceae	Decreases serum albumin during the acute stage, indicating a decrease in protein metabolism; inhibits intracellular enzymes of *T. gondii* and oxidative stress.	El‐Hamid et al. [222]
*Cinnamomum burmannii* (Nees and T. Nees) Blume	Lauraceae	Decreases the average number and diameter of *T. gondii* tissue cysts in infected mice.	Alanazi and Almohammed [224]
*Cinnamomum camphora* (L.) J. Presi	Lauraceae	Inhibits parasite growth by damaging the parasite membrane, disrupts the mitochondrial electron transport chain, and inhibits enzymatic pathways needed for *T. gondii* maturation.	Benoit‐Vical et al. [219]
*Cleome droserifolia* Franch.	Capparaceae	Strong anti‐*T. gondii* activity with an IC_50_ of 12.5 *μ*g/mL and exceptionally high selectivity indices, indicating potent parasite inhibition with low host‐cell toxicity. Contains terpenoids, flavonoids, phenolics, and alkaloids that can interfere with protozoan growth by damaging membranes, inhibiting key enzymes, and modulating oxidative stress. *C. droserifolia* reduced *T. gondii* infection rates to 6.8%, showing strong extracellular parasite killing.	Abdou et al. [235]
*Curcuma longa* L.	Zingiberaceae	Controls oxidative stress, increases GPx and SOD levels, and delays *T. gondii*‐induced liver damage; increases IL‐1b and IFN‐*γ* mRNA genes and improves host survival by boosting the immune system. Mechanisms involve NO activation and apoptosis induction in host cells.	Ezzatkhah et al. [226]
*Jatropha curcas* L.	Euphorbiaceae	Reduces the number of tachyzoites in the contaminated cells with *T. gondii.* Leverages bioactive protease inhibitors and diterpenes to disrupt the parasite lifecycle and structural integrity.	Soares et al. [237]
*Lepidium perfoliatum* L.	Brassicaceae	Inhibits growth of *T. gondii* tachyzoites in infected cells, disrupts tachyzoite cellular division, triggers parasite morphological changes, and alters the host′s cellular defense.	Montazeri et al. [17, 18]
*Lepidium sativum* L.	Brassicaceae	Inhibits the direct growth of infected host cells, leading to growth inhibition of *T. gondii* cells. Also disrupts the parasite′s replication cycle within the host.	Montazeri et al. [17, 18]
*Lycopodium clavatum* L.	Lycopodiaceaea	Core phytoconstituents, lycopodine and apigenin, delay the conversion of tachyzoites to bradyzoites in the brain at 30 days postinfection (dpi), with a significant increase at 60 dpi. Downregulates inflammatory cytokines that delay the conversion to bradyzoites.	Pereira et al. [238]
*Nigella sativa* L.	Ranunculaceae	Increases survival rate and decreases parasite density in liver and spleen. The bioactive metabolite, thymoquinone, upregulates proinflammatory cytokines such as IFN‐*γ*, increasing survival rates and clearing parasites in target organs.	Rasha et al. [239 and Rayan et al. [240]
*Olea europaea* L.	Oleaceae	Inhibits cyst development and has antigranuloma, antiapoptosis, and antinecrosis properties. Active polyphenols, oleuropein and hydroxytyrosol, target cyst development, tissue inflammation, and parasite cell death by inhibiting proinflammatory cytokines and NF‐*κ*B and PI3K/Akt pathways.	Jiang et al. [229]
*Piper betle* L.	Piperaceae	Inhibits proliferation of *T. gondii* cells and reduces parasite invasion of healthy tissues. Extracts like hydroxychavicol and eugenol inhibit proliferation and block invasion of healthy tissues.	Leesombun et al. [232]
*Pleopeltis crassinervata* T. Moore	Poplypodiceae	Inhibits oxygen uptake and oxidative phosphorylation processes of the parasite. Its extract contains a diterpene alcohol, a hexadecenoic acid methyl ester, and various flavonoids that serve to systematically disrupt the structural and metabolic network of the parasite.	Anacleto‐Santos et al. [231]
*Sophora flavecens* Aiton	Fabaceae	Active compounds like matrine and oxymatrine inhibit inflammatory responses and reduce tachyzoites in infected mice and improve hepatic enzymes, such as ALT, AST, GSH, and MDA, through downregulation of the p38 MAPK signaling axis and oxidative stress.	Zhang et al. [30]
*Scutellaria baicalensis* Georgi	Lamiaceae	Has flavonols like baicalin and baicalein, which exhibit antioxidative and free radical scavenging properties, inhibit nitric oxide, and are most effective at inhibiting lipid peroxidation.	Huang et al. [241]
*Satureja khuzestanica* subsp. montana	Lamiaceae	Decreases average tachyzoite burden in infected mice; has antifungal, antinociceptive, and anti‐inflammatory properties, as well as proton exchangers, which reduce the gradient through the cytoplasmic wall; breaks down the proton motive force and reduces the ATP pool, eventually leading to cell death.	Mahmoudvand et al. [236]
*Trichodesma africanum* (L.) Sm	Boraginaceae	*T. africanum* reduced infection rates to 9.6% in vitro, indicating that its constituents impair tachyzoite viability and host‐cell invasion, with strong antibacterial activity, suggesting a mechanism involving membrane disruption and interference with essential biosynthetic pathways.	Abdou et al. [235]
*Vernonia colorata* Drake	Asteraceae	Bioactive compounds such as vernolide and vernodalin target critical parasite processes such as heme biosynthesis and microtubule formation, inhibiting *T. gondii* growth at concentrations that are nontoxic for other cell cultures and eventually achieving parasitic cell death.	Benoit‐Vical et al. [219]
*Vachellia tortilis* (Forssk.) Galasso and Banfi	Fabaceae	Exhibits notable anti‐*T. gondii* activity with an IC_50_ of 24.5 *μ*g/mL, along with high selectivity indices indicating low cytotoxicity. Contains polyphenols, tannins, and flavonoids, capable of inhibiting protozoan parasites by inducing oxidative stress, blocking nutrient uptake, and interfering with parasite replication. Involves disruption of tachyzoite membrane integrity and inhibition of intracellular proliferation.	Abdou et al. [235]
*Zingiber officinale* Roscoe	Zingiberaceae	Inhibits the activation of apoptotic proteins (p53, p21, caspase‐3, and Bax) caused by *T. gondii* proliferation in infected host cells. Its phenolic compounds, like 6‐gingerol and 6‐shogaol, exhibit potent antiparasitic and anti‐inflammatory properties.	Choi et al. [234]

## 10. Potential Application of AI for Detection of Toxoplasmosis

The diagnostic burden of toxoplasmosis falls hardest on regions where the disease is most prevalent and where trained microscopists and specialized equipment are scarcest. This mismatch between where expertise is concentrated and where infection risk is highest is precisely the gap that AI is being explored to close by improving diagnostic efficiency, reducing costs, and expediting *Toxoplasma* detection without requiring the skilled clinicians that microscope‐based methods demand (Table 4). Deep learning models, especially one‐stage and two‐stage detectors, significantly enhance the accuracy and speed of parasite recognition in toxoplasmosis detection, surpassing traditional methods [32, 242, 243].

**Table 4:** AI tools and models applied in the detection and diagnosis of toxoplasmosis.

AI tool/model	Application in toxoplasmosis detection	Reported performance metrics	Reference(s)
ExtremeNet	Detects *Toxoplasma*, *Trypanosoma*, and *Babesia* based on microscopic morphology; limited by parasite overlap and clustering	*Toxoplasma*: AP50 68.47%; AP75 22.60%; AP(0.5:0.95) 30.91%.	Jiang et al. [242] and Zhou et al. [244]
GFS‐ExtremeNet	Improves ExtremeNet using geometric feature spectrum analysis for accurate detection and enumeration of clustered *Toxoplasma*	*Toxoplasma*: AP50 69.77%; AP75 23.50%; AP(0.5:0.95) 32.70%.	Jiang et al. [242] and Zhou et al. [244]
FCGAN	Identifies crescent‐ and banana‐shaped *T. gondii* tachyzoites using unlabeled stained and unstained microscopy images	FCGAN+banana: Accuracy 93.1% (T400) and 94.0% (T1000); *F*1 0.939/0.939; recall 0.960/0.929; precision 0.919/0.949.	Li et al. [32], Hu et al. [245], and Zhang et al. [30]
ResNet18 (modified)	Classifies ocular toxoplasmosis from fundus images by differentiating healthy and inflamed eyes	Accuracy 93.75%; sensitivity 93.75%; specificity 93.75% in the reported ocular toxoplasmosis test evaluation.	Parra et al. [246]
DCTL/CycleGAN‐based DCTL	Enables multiparasite detection of *Toxoplasma*, *Plasmodium*, *Babesia*, and erythrocytes using shape‐guided microscopic analysis	Overall accuracy 95.7%; *F*1 0.957; recall 0.957; precision 0.958; overall AUC 0.995. *Toxoplasma*‐class AUC 0.997.	Li et al. [32] and Hu et al. [245]
VGGNet	Supervised CNN benchmark for evaluating parasite recognition performance against FCGAN and DCTL. Also incorporated into improved COMI for feature extraction.	T400: Accuracy 98.7%, recall 99.6%, and precision 98.2%. T1000: Accuracy 99.1%, recall 99.0%, and precision 99.1%.	Li et al. [32] and Feng et al. [247]
ResNet	Benchmark supervised CNN used for comparison with FCGAN and DCTL in parasite detection performance.	T400: Accuracy 98.4%, recall 98.2%, and precision 99.0%. T1000: Accuracy 98.9%, recall 98.4%, and precision 99.5%.	Li et al. [32]
COMI	Uses CycleGAN and CNN‐based image restoration to correct out‐of‐focus microscopy images and improve parasite detection	Image‐reconstruction metrics: PSNR 38.62; SSIM 0.8951; PCC 0.9726. These are enhancement/reconstruction metrics, not diagnostic accuracy.	Wang et al. [248], Zhang et al. [30], and Feng et al. [247]
CycleGAN	Used in FCGAN, DCTL, and COMI for image translation, image enhancement, and parasite recognition without extensive labeled datasets.	No standalone toxoplasmosis diagnostic accuracy reported for CycleGAN itself in the cited sources; used as an image‐translation/enhancement component.	Wang et al. [248] and Feng et al. [247]
AutoML	Provides a code‐free platform for ocular toxoplasmosis diagnosis and lesion detection from fundus images	Binary ocular toxoplasmosis model: AuPRC 0.945; sensitivity 100%; specificity 83%; accuracy 93.5%. Lesion detection: AuPRC 0.600; precision 93.3%; recall 56%.	Milad et al. [249]

ExtremeNet has been utilized in the detection of unicellular protozoans such as *Toxoplasma*, *Trypanosoma*, and *Babesia* sp. based on their shapes under a microscope, achieving average precision scores of 86 and 88 at Intersection over Union (IoU) thresholds of 0.75 and 0.50, respectively (H. [242, 244]). It struggles, however, to enumerate parasites that clump together and overlap in layers. Geometric‐feature spectrum (GFS)‐ExtremeNet was developed to correct this weakness: By applying an integrated GFS at the processing stage, it can separate and count clumped unicellular protozoans, achieving improved average precision scores of 87 and 90 at IoU thresholds of 0.75 and 0.50, respectively, in diagnosing toxoplasmosis [242, 244].

Li et al. [32] took a transfer learning approach grounded in three characteristic shapes of *T. gondii*, the crescent, banana, and star forms, to design a microscopic–macroscopic associated object pulling system paired with a fuzzy cycle generative adversarial network (FCGAN) for recognizing crescent‐ or banana‐shaped *T. gondii* tachyzoites, a stage central to diagnosing and controlling acute toxoplasmosis [245]. The FCGAN model built around the banana shape outperformed unsupervised methods and approached the accuracy of supervised models such as ResNet, Visual Geometry Group Network (VGGNet), and GoogleNet. Because FCGAN requires no labeling of *T. gondii* and still matched supervised deep learning methods while achieving greater accuracy across both stained and unstained parasites, it stands out as a particularly promising diagnostic tool for settings where expert annotation is not readily available [32]. This method reached detection accuracy and precision of 93.1% and 94.0% for microscopic images of *Toxoplasma* at ×400 and ×1000 magnifications, respectively [30, 32].

In ophthalmology, Parra et al. [246] refined a ResNet18 neural network for automatic detection of ocular toxoplasmosis, replacing its final layer with a softmax layer containing two artificial neurons to sharpen classification accuracy. By training and evaluating the model on 160 fundus images captured by expert ophthalmologists, they achieved a sensitivity of 94% and specificity of 93% in distinguishing healthy eyes from those with active inflammation, results that meet the standards applied to retinopathy screening more broadly, such as diabetic retinopathy assessment [250].

Most AI parasite detection tools remain narrowly built for a single parasite, leaving multiparasite detection underdeveloped. Li et al. [32] partially addressed this limitation by drawing on expert knowledge of parasite morphology to build a CycleGAN‐based deep cycle transfer learning (DCTL) system capable of identifying multiple apicomplexan parasites, including *Toxoplasma*, *Plasmodium*, and *Babesia* [245]. The model uses macroscopic reference shapes—ring, banana, pear, and apple—to guide microscopic image analysis and detect *Toxoplasma*, *Plasmodium*, *Babesia*, and erythrocytes, respectively, reaching an average accuracy of approximately 96% and precision of 0.96. DCTL also outperformed three supervised deep CNNs, VGGNet, ResNet, and GoogleNet, across accuracy, precision, *F*1‐score, and recall [32]. A single tool capable of screening for several parasites at once is especially valuable in low‐resource settings, where the cost of deploying separate diagnostic systems for each pathogen is often prohibitive.

Image quality remains a persistent constraint on these systems. Diffraction limits in optical microscopes make consistently sharp resolution difficult to achieve, leaving many images blurred and degrading the performance of AI detection tools built on them [241]. Correcting out‐of‐focus microscopic images (COMI) is a deep learning method that combines CycleGAN with multilayer CNNs to restore focus in images from both bright‐field and confocal fluorescence microscopy. Using regression mapping, it converts out‐of‐focus images of protozoan parasites, including *Toxoplasma*, into in‐focus versions, improving downstream detection, although checkerboard artifacts in zoomed‐in images remain a limitation [30, 247, 248]. This kind of image restoration carries particular weight for decentralized diagnostics: It allows lower‐grade or poorly maintained microscopy equipment, the kind more likely to be found in under‐resourced clinics, to still generate usable diagnostic images rather than requiring costly high‐end instrumentation as a precondition for accurate detection.

To address weaknesses across detection methods more broadly, Feng et al. [247] drew on expert knowledge to improve classification, detection, and out‐of‐focus correction for parasites such as *Toxoplasma* and *Trypanosoma*, refining DCTL, GFS‐ExtremeNet, and COMI for this purpose. The revised DCTL incorporates two additional feature extractors, and the revised COMI adds two pretrained VGGNets from ImageNet for feature extraction and content loss computation, with a geometric spectrum integrated at postprocessing for further precision gains. The refined protocol produced better results overall, though the gains for DCTL and GFS‐ExtremeNet were modest relative to their original versions, whereas COMI showed a somewhat larger improvement [242, 247].

The most consequential recent development for cost reduction and decentralization may be the emergence of code‐free AI models, which let parasitologists without programming expertise build their own detection tools. Milad et al. [249] developed a code‐free automated machine learning (AutoML) system comprising a binary model for distinguishing ocular toxoplasmosis from healthy eyes and an object detection model for visualizing toxoplasmosis lesions. Benchmarked against expert‐designed models using 304 labeled fundus images, the AutoML system achieved an area under the precision–recall curve of 0.945, 100% sensitivity, 93.5% accuracy, and 83% specificity, outperforming the custom‐built alternatives. Because such systems remove the need for in‐house machine learning expertise or dedicated software engineering teams, they represent a realistic path toward placing diagnostic capacity directly in clinics and laboratories in high‐prevalence, low‐income regions rather than routing all diagnostic decisions through centralized reference centers. Combined with image‐restoration approaches like COMI, which reduce dependence on high‐specification imaging hardware, code‐free AutoML tools point toward a diagnostic model in which both the software and the equipment barriers that have historically concentrated toxoplasmosis expertise in well‐resourced settings are substantially lowered, narrowing the gap between where the disease burden is greatest and where the capacity to detect it currently resides.

## 11. Conclusion and Future Directions

Toxoplasmosis continues to impose a substantial and unevenly distributed burden on global health, driven by the parasite′s ecological versatility, its entrenchment in both human and animal reservoirs, and the socioeconomic conditions that shape exposure risk. The persistence of this burden is not simply a biological problem but a structural one: Current management still relies on a narrow set of antiparasitic drugs that have changed little in decades, carry significant toxicity, and are increasingly compromised by resistance. Meanwhile, diagnostic infrastructure in many of the regions most affected by *T. gondii* remains too costly, too centralized, or too slow to support timely intervention. Addressing toxoplasmosis at scale therefore demands more than incremental refinement of existing tools; it requires a deliberate paradigm shift toward alternatives that are both biologically effective and economically viable for the populations that need them most (Figure 3).

**Figure 3:**
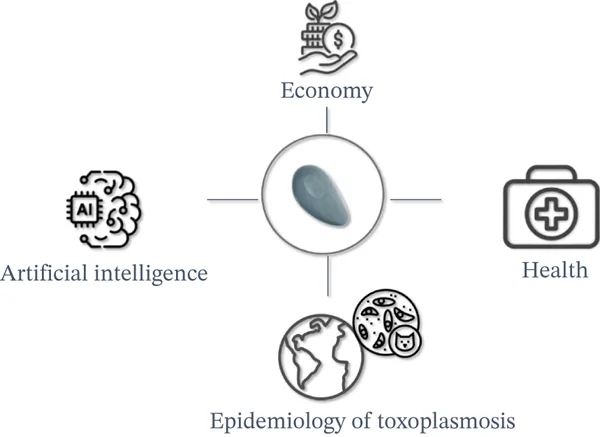
Impact of toxoplasmosis on artificial intelligence, epidemiology, health, and economy. *T. gondii* infection can influence behavior and cognitive functions, which may have implications for AI systems designed to model human behavior, as infected individuals may exhibit personality changes and riskier behavior [251]. Latent toxoplasmosis has been linked to various psychiatric disorders, including schizophrenia and depression [252, 253]. The presence of the parasite in the brain may lead to neurotransmitter imbalances, contributing to these mental health issues [251]. Cognitive impairments associated with latent toxoplasmosis may affect productivity in the workforce. Individuals with decreased cognitive function or increased risk‐taking behavior could lead to higher accident rates and lower overall workplace productivity [251, 253]. *T. gondii* is prevalent worldwide, with seroprevalence rates varying significantly by region. Understanding these epidemiological patterns is crucial for developing targeted public health interventions.

Botanical therapeutics represent one pillar of this shift. Plant‐derived compounds acting on parasite‐specific pathways, including folate metabolism, electron transport, and the tachyzoite–bradyzoite interconversion that underlies chronic infection, offer a route to treatments that are less toxic, more accessible, and less dependent on the pharmaceutical supply chains that limit drug availability in low‐resource settings. Realizing this potential will require sustained investment in validating efficacy and safety, but the biological rationale is already well established and warrants prioritization rather than treatment as a secondary research track.

The second pillar is diagnostic transformation through AI. AI‐driven screening tools can lower the cost and technical burden of detection, extend surveillance into settings that lack specialist infrastructure, and support the kind of large‐scale, standardized testing that current fragmented data collection cannot provide. Pairing such tools with genomic profiling of circulating *T. gondii* strains would allow diagnostic and treatment strategies to be matched to actual patterns of virulence and drug resistance rather than applied uniformly, whereas parallel investigation of host–pathogen interactions at the molecular level would help identify the immune‐evasion mechanisms that sustain chronic infection and expose new therapeutic targets.

These two pillars are mutually reinforcing rather than separate initiatives. Affordable AI‐based screening makes it possible to identify infections early enough for botanical treatments to be deployed effectively, whereas data generated through expanded testing can, in turn, refine both diagnostic algorithms and the therapeutic targets prioritized in drug discovery. Achieving this integration will depend on coordination across medicine, environmental science, and public health policy, including standardized veterinary testing protocols and, where justified by the scale of livestock exposure, regulatory frameworks that move surveillance from voluntary recommendation to enforceable practice. A strategy built on these terms offers a more realistic path to reducing the global burden of toxoplasmosis than continued reliance on the conventional tools that have so far proven insufficient to the scale of the problem.

## Author Contributions

A.F.A.: writing—original draft and review and editing. W.E., J.H.N.O., A.A‐A., A.A., C.N.L.T‐M., B.K.G., S.A.A., J.A.M.K.S., and J.A.A.Y.: writing—original draft. A.K.D.: study conceived and designed, writing—original draft and review and editing.

## Funding

No funding was received for this manuscript.

## Conflicts of Interest

The authors declare no conflicts of interest.

## Data Availability

All the data generated during the study can be found in this manuscript.
